# Myelodysplasia and Mast Cell Leukemia with t(9;22)

**DOI:** 10.1155/2017/9249302

**Published:** 2017-11-26

**Authors:** Kathryn J. Lago, Matthew P. Shupe, William N. Hannah, Gopalrao V. N. Velagaleti, Christina Mendiola, Veronica Ortega, Brian R. Haney

**Affiliations:** ^1^Internal Medicine Service, San Antonio Military Medical Center, Fort Sam Houston, San Antonio, TX, USA; ^2^Hematology and Oncology Service, San Antonio Military Medical Center, Fort Sam Houston, San Antonio, TX, USA; ^3^Department of Pathology, University of Texas Health Science Center, San Antonio, TX, USA

## Abstract

**Introduction:**

Mast cell leukemia (MCL) is a rare variant of systemic mastocytosis. Most cases of mast cell leukemia do not have cytogenics performed. Furthermore, there is no consistent chromosomal abnormality identified in MCL. This is the first reported case of MCL with a (9;22) translocation.

**Case Report:**

An 80-year-old female presented with pancytopenia and was diagnosed with MDS. Over time, she required hospitalizations for platelet transfusions with increased frequency. She developed fatigue and weakness along with gastrointestinal symptoms. On exam, she had diffuse abdominal tenderness and a maculopapular rash. Her lab results revealed a new basophilia. A bone marrow biopsy showed 100% cellularity with many aggregates of mast cells. Chromosomal analysis showed t(9;22) with confirmed BCR/ABL1 fusion by fluorescence in situ hybridization (FISH).

**Discussion:**

MCL has a poor prognosis due to the aggressive nature of the disease and ineffective therapies. Translocation (9;22) is known to be associated with MDS transformations to acute leukemia; however, this translocation has never been reported in MCL. Further research on the relationship between t(9;22) and MCL could lead to development of improved therapeutic options.

## 1. Introduction

Mast cell leukemia (MCL) is a rare, aggressive form of systemic mastocytosis (SM) representing less than 0.5% of all mastocytosis cases [1]. In addition to meeting the 2008 WHO criteria for systemic mastocytosis [2], a diagnosis of MCL requires twenty percent or greater bone marrow infiltration by atypical mast cells or greater than ten percent circulating mast cells in the peripheral blood [3]. Myelodysplastic syndrome (MDS) transforming into MCL has been reported in less than ten cases in the literature. Due to the rarity of this disease, there are limited data regarding cytogenetic abnormalities and molecular characteristics of those diagnosed with MCL [1]. The most well-studied mutations in MCL involve the *KIT* gene, which is a somatic mutation of the protooncogene that encodes the receptor for stem cell factor (SCF) [4]. Approximately 50% of cases of MCL have cytogenetic analysis performed with the majority of these cases showing normal cytogenetics [1]. In this case report, we describe the first published case of MCL-MDS with a (9;22) translocation.

## 2. Case Report

An 80-year-old female presented in 2012 with pancytopenia, and upon further workup, she was diagnosed with myelodysplasia with excess blasts-2. Her initial bone marrow biopsy showed dysplasia in the erythroid and megakaryocyte lineages with 10–12% blasts without any reported mast cell. A serum tryptase was not obtained at this time. Chromosome analysis from the bone marrow aspirate showed 16 of the 20 cells analyzed with a 20q deletion with the karyotype 46,XX,del(20)(q11.2q13.1)[16]/46,XX[20] [5]. Fluorescence in situ hybridization analysis was carried out using the AML/MDS panel consisting of probes to detect monosomy 5/5q deletion, monosomy 7/7q deletion, trisomy 8, monosomy 20/20q deletion, MLL gene rearrangement, t(8;21), t(15;17), and inv(16) (Cytocell UK Ltd., Windsor, CT). Results were normal for most probes except for chromosomes 7 and 20. Interphase FISH analysis showed monosomy 7 with probes for *RELN* (7q22 labeled with spectrum orange) and *TES* (7q31.2 labeled with spectrum green) in 9.5% of the nuclei, and a 20q deletion with probes for *PTPRT* (20q12q13 labeled with spectrum orange) and *MYBL2* (20q13.12 labeled with spectrum green) was seen in 58.5% of the nuclei. She was started on azacitidine at this time of her initial diagnosis.

Over the course of two years, she required frequent hospitalizations for platelet transfusions. A follow-up cytogenetic evaluation in 2013 showed only 20q deletion on both chromosome (20/20 cells) and FISH (91.5% of interphase cells) analyses. In 2014, after 18 cycles of azacitidine, she developed fatigue, weakness, anorexia, diffuse abdominal pain, nausea, and vomiting. On physical exam, she had diffuse abdominal tenderness to palpation worse in the midepigastrium. Additionally, she had a faint maculopapular rash on her back, arms, and legs with significant excoriations. Her lab results revealed stable pancytopenia with a WBC count of 1.4 × 10^3^ and a hemoglobin level of 11.2 g/dL. However, she was becoming increasingly dependent upon platelet transfusions. Her automated differential showed a relative excess of basophils. Given her increased frequency of platelet transfusions, relative excess of basophils, and constitutional symptoms, a peripheral blood smear and bone marrow biopsy were examined.

**Figure 1:**
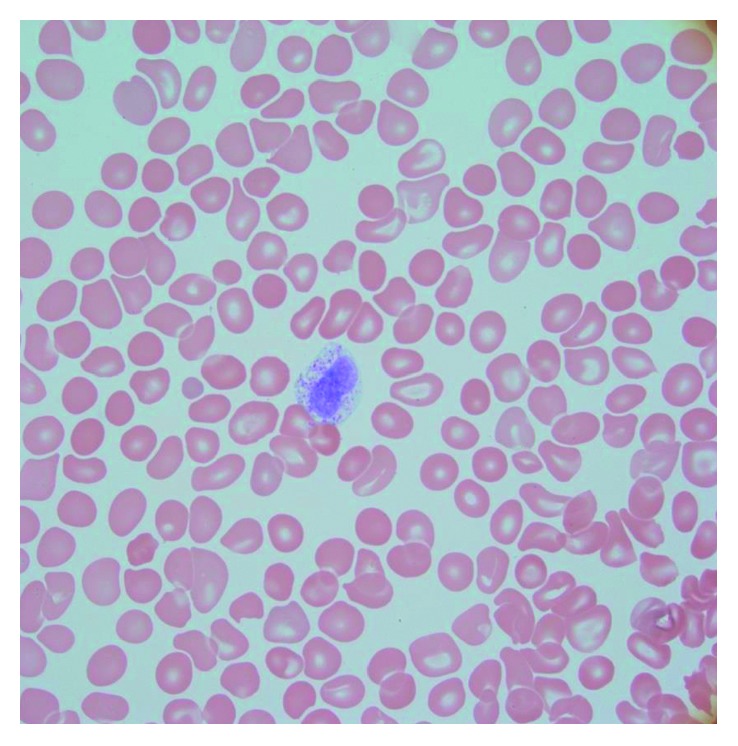
Peripheral smear with mast cell.

**Figure 2:**
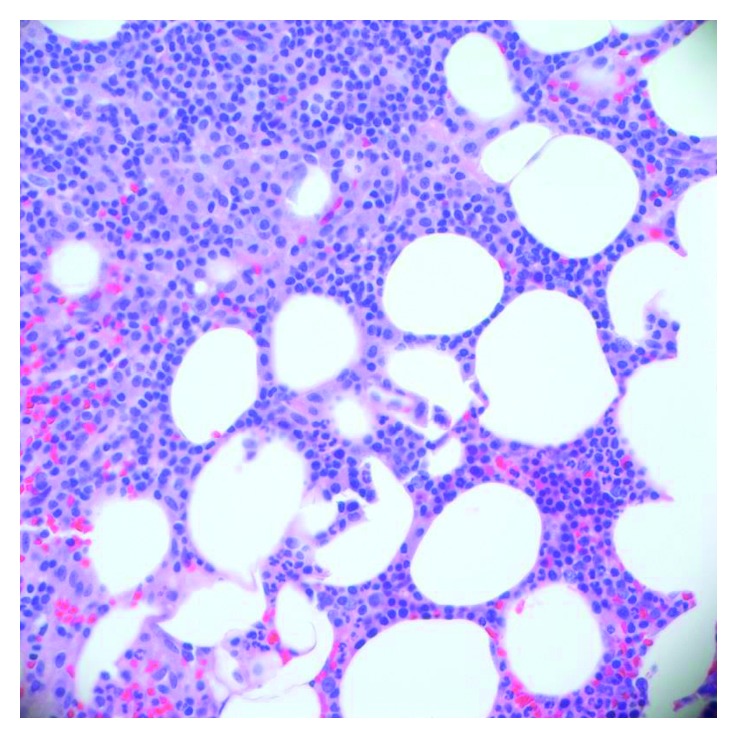
Hypercellular bone marrow with aggregates of mast cells.

**Figure 3:**
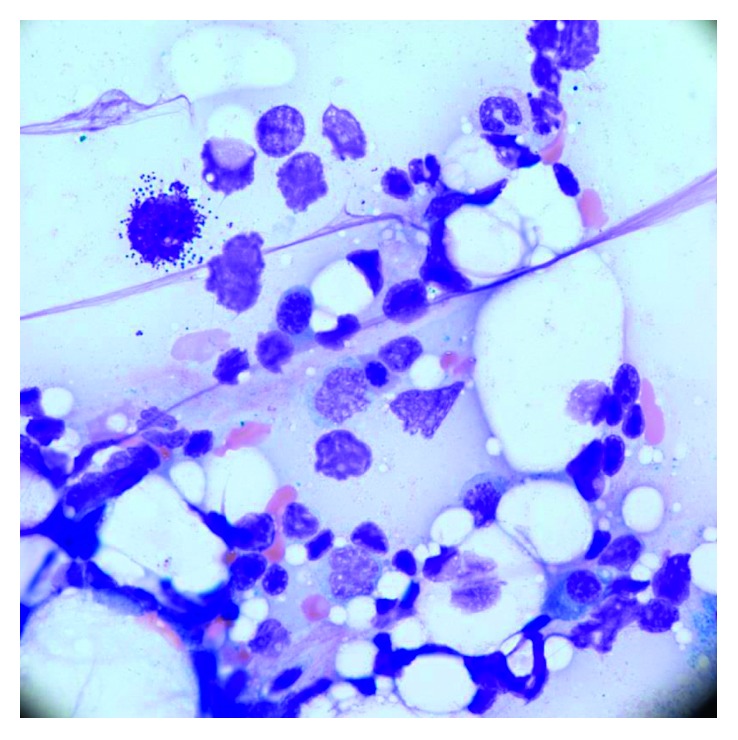
Bone marrow aspirate with degranulated mast cell.

**Figure 4:**
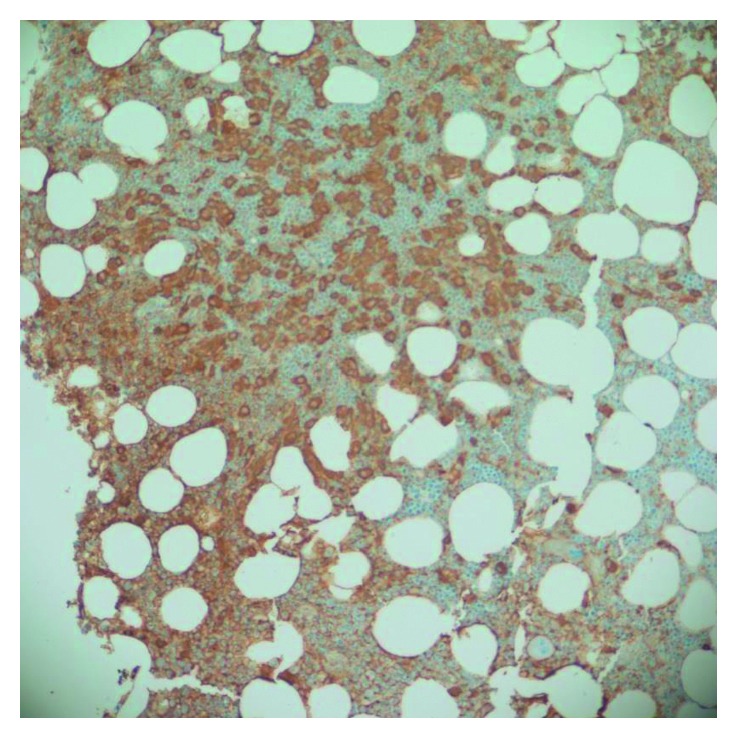
Tryptase stain of bone marrow.

The peripheral blood smear showed 12% mast cells (Figure 1). Her bone marrow biopsy showed 100% cellularity with aggregates of interstitial, perivascular, and paratrabecular mast cells in fibrotic stroma with spindling (Figure 2). The bone marrow aspirate showed 10% myeloid blasts and 20% mast cells. The mast cells showed degranulation with monolobated nuclei and some having blast-like chromatin (Figure 3). Immunohistochemistry stains showed CD117 (c-KIT) positivity, highlighting the aggregates of mast cells as well as individual mast cells. There was also CD2 and CD25 positivity seen in the aggregates of mast cells. A tryptase stain was diffusely taken up in the bone marrow (Figure 4). Flow cytometry on the peripheral blood and bone marrow showed aberrant CD117, CD25, CD33, CD45, and CD13 expressions. Subsequently, a serum tryptase level was found to be 600 mcg/L (2.2–13.2 mcg/L). Therefore, she fulfilled the diagnostic criteria for MCL with her peripheral smear and bone marrow findings, grossly elevated serum tryptase, and CD2/CD25 markers. Despite her CD117 positivity seen in mast cells on the immunohistochemical stain, c-KIT mutational analysis was not performed.

**Figure 5:**
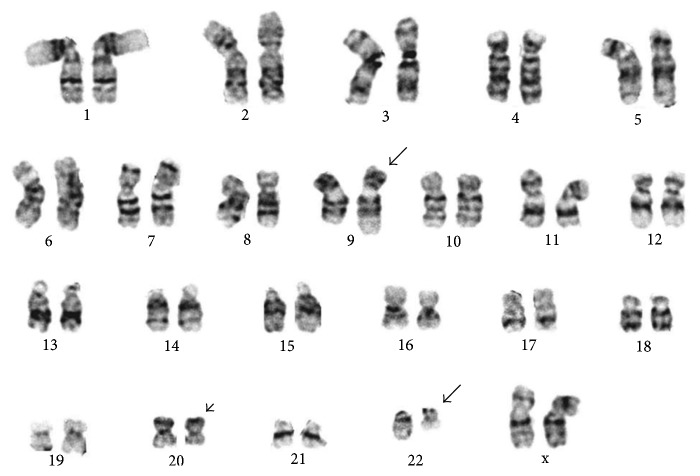
Karyotype from the 2014 bone marrow aspirate after diagnosis of transformation to mast cell leukemia. The arrows point to the t(9;22) and arrowhead to the del(20q).

**Figure 6:**
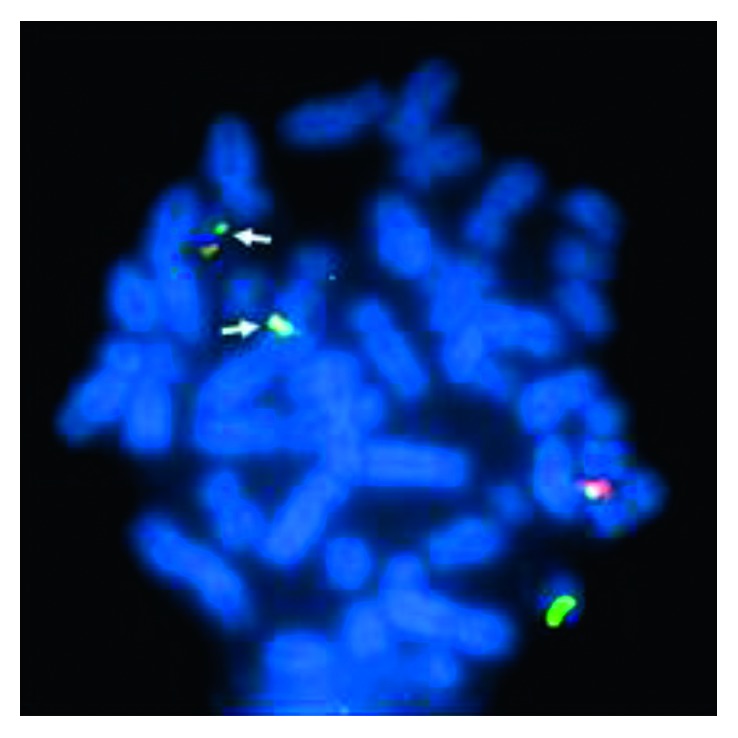
Metaphase with FISH for ASS1 (spectrum aqua)/ABL1 (spectrum orange)/BCR (spectrum green). The arrows point to the 2 fusion signals on der(9) and der(22). The other fusion signal on normal chromosome 9 is the result of juxtaposition of ASS1 (spectrum aqua) with ABL1 (spectrum orange) and does not represent the typical fusion seen with BCR/ABL1.

Once the diagnosis of MCL was established, a bone marrow aspirate was sent for chromosomal analysis to further evaluate her disease process. Her karyotype showed that 17 of the 20 cells had the 20q deletion seen previously. However, 7 of the 17 cells also showed the characteristic t(9;22) (Figure 5). The karyotype was interpreted as 46,XX,del(20)(q11.2q13.1)[10]/46,idem,t(9;22)(q34;q11.2)[7]/46,XX[3] [5]. Since t(9;22) has never been reported in MCL, additional FISH analysis was carried out with the BCR/ABL plus translocation dual fusion (3 color) probe (Cytocell UK Ltd., Windsor, CT) consisting of probes for ASS1 (9q34.1 labeled in spectrum aqua), ABL1 (9q34.1 labeled in spectrum orange), and BCR (22q11.2 labeled in spectrum green) to confirm this t(9;22) which resulted in the characteristic BCR/ABL1 fusion. Interphase FISH analysis confirmed the presence of BCR/ABL1 fusion in 20% of the nuclei and also demonstrated a 20q deletion in 91.5% of the nuclei (Figure 6). To investigate if the BCR/ABL1 fusion was present from the beginning, FISH studies were carried out using the same BCR/ABL plus translocation dual fusion (3 color) probe on her original bone marrow biopsies from both 2012 and 2013. The result was positive for 20q deletion but negative for BCR/ABL1 (200 interphase nuclei) on both samples, indicating that the BCR/ABL1 fusion was present after transformation to MCL.

The patient's age, comorbidities, and poor performance status made her a poor candidate for stem cell transplantation. Her thrombocytopenia requiring weekly platelet transfusion made her a poor candidate for treatment with midostaurin. Following a discussion with the patient, she opted against tyrosine kinase therapy secondary to the gastrointestinal side effect profile. She was treated palliatively with prednisone 50 mg, ranitidine 150 mg, cetirizine 10 mg, and esomeprazole 40 mg. With the use of these medications her abdominal pain greatly improved to her tolerating a regular diet and her nausea and vomiting completely resolved. She died two months after her initial diagnosis while on hospice care.

## 3. Discussion

The most well-studied and common genetic mutation associated with MCL is a c-KIT mutation. Approximately 50% of MCL cases present with some variants of a c-KIT mutation [1]. The most common c-KIT mutation is the D816V in exon 17 of the tyrosine kinase domain 2 and conveys inherent imatinib resistance [6]. Newly found cytogenetic abnormalities in MCL may lead to new therapeutic opportunities for targeted agents.

Less than half of reported MCL cases include cytogenetic profiles. In these cases, only a few unique cytogenetic abnormalities have been identified. These include t(10;16) and t(8;21) which were individually seen in de novo cases of MCL [1]. 5q deletions have been associated with MCL-MDS. In these cases, the 5q deletion was thought to be associated with the previously diagnosed MDS [1].

The t(9;22) is a common translocation seen in ALL, AML, and CML. The discovery of the Philadelphia chromosome revolutionized the treatment of leukemia with the development of targeted tyrosine kinase inhibitors. Furthermore, detection of the Philadelphia chromosome in the abovementioned leukemias has been used as a prognostic tool [7]. However, the Philadelphia chromosome does not always represent evolution to a chronic leukemia or blast crisis.

A case of acute basophilic leukemia with a t(9;22) has been described in the literature. Initially, it was thought that this case represented CML with secondary basophilic leukemia. However, the case had no history of CML and had distinct features of basophilic leukemia. Therefore, it was determined that this case represented a basophilic leukemia with a t(9;22) and not just a sequela of CML [8]. Similarly, our reported case of MCL with a t(9;22) may represent a distinct form of MCL as opposed to an AML/CML evolution. The fact that the t(9;22) was not present in the original bone marrow aspirates or follow-up bone marrow aspirates suggests that this translocation was acquired at the time of her presentation with MCL. The largest limitation of our case is that we did not demonstrate that the 20q deletion was confined to myeloid lineage and the t(9;22) was seen in mast cells. Lack of the sample makes it not possible to perform sequential studies to first confirm the identity of cell lineages with immunostaining and following FISH studies to demonstrate the presence or absence of these clonal abnormalities in the respective cell lineages. Our interphase FISH studies on the original bone marrow aspirates showed the absence of BCR/ABL1 fusion, indicating that the t(9;22) originated at the time of presentation of MCL. Alternately, the Philadelphia chromosome that was present in the 7/17 cells with the persistent del(20q) could represent a subclone. While it is possible that there are BCR/ABL1 fusion positive cells in these original bone marrow aspirates, the numbers were not high enough for FISH studies to identify them. A PCR-based assay for detecting the BCR/ABL1 transcripts on these original aspirates would have conclusively shown whether the transcripts are present, but lack of fresh material currently precludes such PCR-based studies.

Systemic mastocytosis is associated with 30% of hematological neoplasms [9], and thus, the classification of systemic mastocytosis with associated hematological neoplasm (SM-AHN) exists [10]. Few cases of SM-CML have been reported in the literature. Hussein et al. [9] describe a BCR-ABL-positive CML that meet the major criteria for systemic mastocytosis and the minor criteria of CD117 (KIT) expression. In this case, SM criteria and t(9;22) were present at the time of diagnosis. The FISH analysis showed that the t(9;22) belonged solely to the leukocytes and megakaryocytes (the CML cells) and not to the mast cells. The Hussein et al. [9] case does highlight the largest limitation to our case presentation in that we were not able to identify the t(9;22) solely in the newly arisen mast cell lineage. Importantly, the abovementioned case is distinctly different from our reported case as it does not meet the criteria for mast cell leukemia and there is no evidence of prior hematologic neoplasm. Thus, our case distinctly represents MCL-MDS with a t(9;22).

Mast cell leukemia is a rare disease with poor prognosis and limited treatment options. The median survival for this aggressive disease is 6 months [1]. While the most common genetic mutation in MCL, c-KIT, confers imatinib resistance, the demonstration of other targetable mutations may lead to new therapeutic options. Thus, it is important to report all cases with new unique cytogenetic features in hopes of developing better therapeutic options that prolong patient survival. This is the first reported case of MCL-MDS with a t(9;22).
